# Malaria, anaemia and nutritional status among schoolchildren in relation to ecosystems, livelihoods and health systems in Kilosa District in central Tanzania

**DOI:** 10.1186/s12889-015-1932-x

**Published:** 2015-06-17

**Authors:** Leonard E.G. Mboera, Veneranda M. Bwana, Susan F. Rumisha, Robert C. Malima, Malongo R.S. Mlozi, Benjamin K. Mayala, Grades Stanley, Tabitha Mlacha

**Affiliations:** National Institute for Medical Research Headquarters, P.O. Box 9653, Dar es Salaam, Tanzania; National Institute for Medical Research, Amani Research Centre, Muheza, Tanzania; Sokoine University of Agriculture, Chuo Kikuu, Morogoro, Tanzania

**Keywords:** Malaria, Anaemia, Nutritional status, Children, Ecosystems, Livelihoods, Tanzania

## Abstract

**Background:**

Malaria prevalence and transmission intensity in Tanzania is heterogeneous with spatial and temporal variations between geographical areas and ecological systems. The objective of this study was to determine the prevalence of malaria, anaemia and nutritional status in relation to livelihoods, ecosystem and health systems in Kilosa District in central Tanzania.

**Methods:**

This study was conducted in four villages, two characterised by rice irrigation ecosystem and the other two by dry savannah ecosystem and pastoral livelihoods. In each ecosystem, one of the villages had a healthcare facility. Schoolchildren were screened for malaria infection using malaria rapid diagnostic test (mRDT) and microscopy and they were assessed for their anaemia and nutritional statuses.

**Results:**

A total of 1,019 school children (age = 4–16 years) were screened for malaria infection. The overall prevalence of *Plasmodium falciparum* infection was 10.6 % and 4.5 % by mRDT and microscopy, respectively. Children from pastoral villages had lower (2.9 %) prevalence of malaria than their counterparts (18.2 %) in the rice irrigation villages. A significantly high risk of malaria was observed among children in rice irrigation than in the pastoral ecosystem (OR: 0.13; 95%CI 0.07, 0.23). Children living in areas with health care facilities had a low odd of malaria infection by 45 % (OR: 0.55; 95 % CI = 0.35, 0.86). Overall, the prevalence of anaemia in the district was 43.4 % (n = 775); and 58.3 % of those with severe anaemia were among children from the pastoral villages. Anaemia was significantly higher among children not using mosquito nets (p = 0.049); and among those with malaria infection (p <0.001). The majority (96 %) of the children had Body Mass Index less than 18.5 kg/m^2^ which indicate high proportion of underweight.

**Conclusion:**

There are significant variations in the risk of acquiring malaria infection between different ecosystems and livelihoods. These findings suggest that malaria control programmes must take into account ecosystems and livelihoods of the targeted population through an integrated management of malaria and nutrition approach.

## Background

Malaria is the single most important and widespread mosquito-borne disease in Tanzania. The disease is endemic in most parts of the country, and remains as a major cause of hospital attendance, admission and death [1]. However, malaria burden in the country is heterogeneous with malaria prevalence rates and entomological inoculation rates varying from one area to another [2–5]. Similarly, there are variations in malaria mosquito vector composition and transmission intensities in localised areas, within districts and even within villages. The significant variations in malaria transmission intensities and prevalence are associated with variations in ecological systems [4–7] and socio-economic factors [8, 9]. In spite of the many past and current efforts to combat malaria, the disease has remained a major public health problem in Tanzania for various reasons including human socio-cultural factors, inequity, weak health systems, limited budgets, poor governance and accountability, antimalarial drug and insecticide resistance, environmental changes and demographic factors [1].

Malaria represents a complex, multi-dimensional health problem with a host of interacting variables. Such complex health problems are difficult to solve without understanding ecosystems, livelihoods and health delivery systems contexts. This is because, health is a social construct negotiated in the context of a better understanding of the constraints and opportunities provided by the three variables of which people are integral parts [10]. Ecosystems and various livelihoods activities have impact on mosquito productivity, mosquito biting exposures and hence malaria transmission intensity [11–14]. For instance, some agro-ecosystems provide favourable conditions for mosquito breeding, and hence higher malaria prevalence [4, 5, 14, 15]. Other livelihood activities that are associated with higher malaria prevalence include nomadic pastoralism, mining, fishing and construction [1]. On the other hand, the availability of health care facilities has been associated with low malaria prevalence in Tanzania [16, 17].

Malaria is mostly a disease of rural communities in Tanzania, where agriculture is the backbone of the household economy. Studies in Sub-Saharan Africa have shown that the tropical climate and land use changes have either a positive or negative effect on mosquito reproduction and survival and exposure of humans to infectious mosquito bites [18, 19]. Crop irrigation which aims to increase productivity and boosts economic growth, has often been associated with increased in mosquito productivity and hence malaria transmission [4, 5, 15]. However, some evidences indicate that in malaria endemic areas, communities involved in crop irrigation benefit from the income generated from increased productivity and thus have greater use of malaria intervention tools and better access to improved healthcare [15]. Thus, livelihoods practices impact on malaria by either increasing or decreasing the risk of the disease.

A good understanding of the nature and dynamics of certain ecosystem and livelihoods variables and their relationship to malaria prevalence and transmission intensity is crucial in identifying and addressing interventions that may reduce malaria while increasing household productivity. This study was, therefore carried out to determine malaria prevalence, anaemia and nutritional status among schoolchildren in areas representing different ecosystems and livelihoods in Kilosa District in central Tanzania.

## Methods

### Study site

The study was carried out in Kilosa District (5°55′ -7°53′S; 36°30′ - 37°30E) in central Tanzania. The district has a total surface area of about 14,400 km^2^ and a population of 489,513 people living in 105,635 households with an average household size of 4.2 people [20]. The district has a tropical climate, characterised by a monomodal rainfall pattern which begins in October with a peak in April-May. The mean annual temperature is 25 °C. Agriculture is the main activity of most people in the district and is characterized by predominance of smallholder and estate farms. The main crops are maize, rice, sorghum, beans, cassava, sweet potatoes, cotton, sunflower, sesame and sisal. Free-range livestock production is also an important land use in the district [14].

**Fig. 1:**
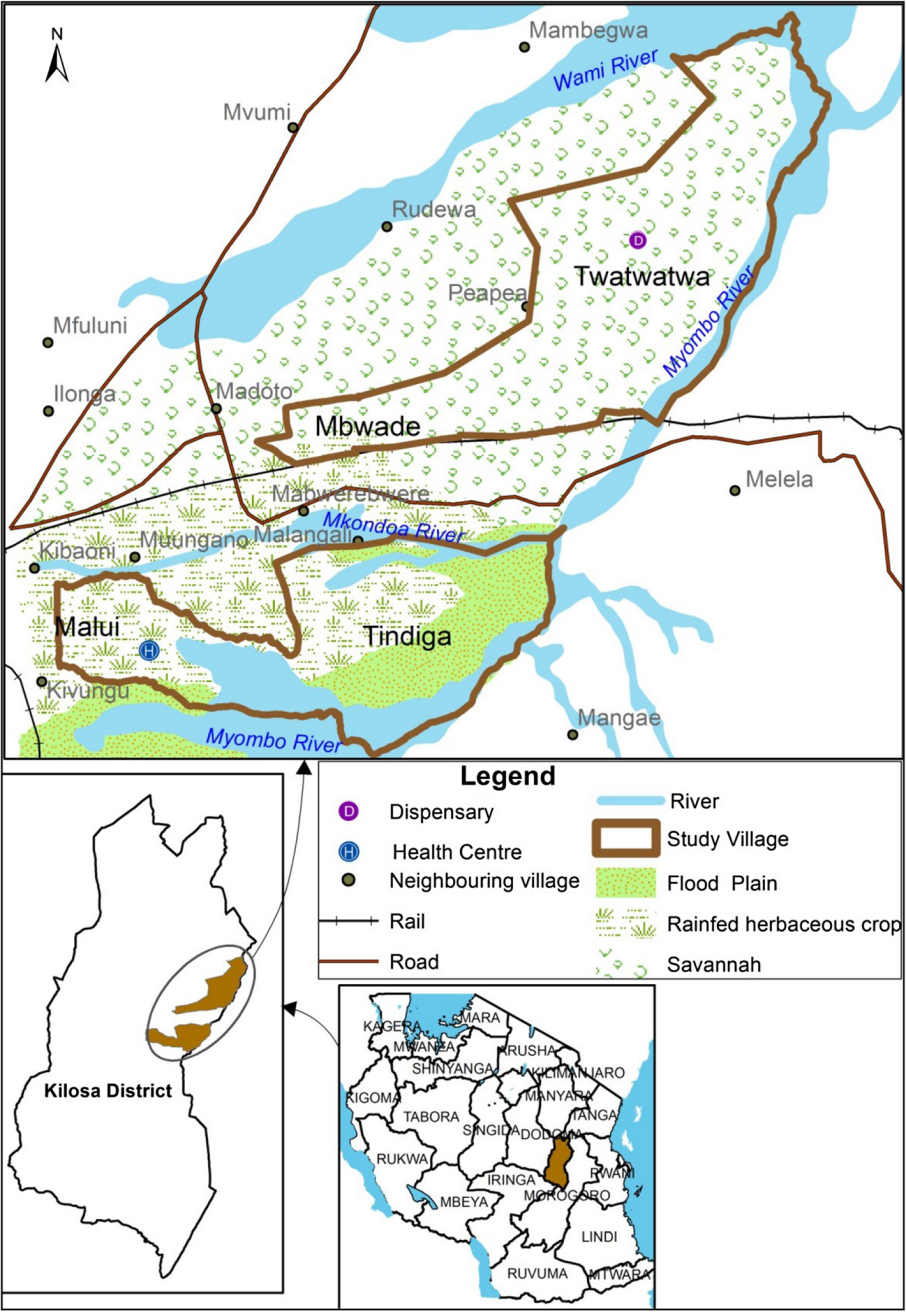
The Study area in Kilosa District, Tanzania

This study was conducted in four villages, namely Tindiga, Malui, Twatwatwa and Mbwade. Tindiga and Malui are in the south-eastern part of the district and characterised by swampy flatland and wetlands lying on the Kilangali alluvial basin. Most of the communities in Tindiga and Malui are small-scale farmers of rice using the traditional ground flooding irrigation practice. Mbwade and Twatwatwa are located in the north-eastern part of the district and are characterised by dry savannah type of vegetation, with most of the areas covered with short grasses, trees and shrubs that provide a wide range of pasture for livestock grazing (Fig. 1). Mbwade and Tindiga villages are inhabited mainly by the Maasai pastoralists keeping cattle, sheep, goats and donkeys. Health facilities (dispensaries) were available at Tindiga and Twatwatwa villages.

### Malariometric and nutritional status surveys

This study was carried out in May 2012 and involved schoolchildren. Each individual child was screened for the presence of malaria parasites using both microscopy and malaria Rapid Diagnostic Test (mRDT) (SD Bioline Malaria Ag. Pf/Pan test kits Device, Gewerbestrasse, Switzerland). Thick and thin blood smears were stained with Giemsa and examined with a binocular microscope with an oil immersion lens to quantify the parasitaemia. Parasitaemia was measured by counting the number of asexual parasites against the number of leukocytes in the blood film, based on a putative count of 8,000 leukocytes per microlitre. The number of asexual parasites was counted against 200 leukocytes using a hand tally counter. A slide was considered negative if no malaria parasite was seen after scanning 200 fields. The blood films were examined by experienced microscopists without reference to the results of the malaria rapid diagnostic test.

A sub-sample of the blood was used to determine haemoglobin concentrations by using a HemoCue haemoglobinometer (HemoCue, Ängelholm, Sweden). All children were examined for splenomegaly through the palpation of the spleen in a horizontal position by the research physician [21] and body temperature was taken. Schoolchildren were interviewed with the assistance of the schoolteachers regarding recent history of fever and treatment. Socio-demographic data such as age, sex, place of residence and use of a mosquito net were recorded.

To determine the nutritional status, the body weight of each child was measured and recorded to the nearest 0.1 kg. Height was measured with a child standing upright using a stadiometer (Leicester, CMS, Campden, UK) and was recorded to the nearest 0.1 cm. The measurement was recorded while the child was standing without shoes on a horizontal flat plate.

### Data analysis

All malariometric data were entered and verified in Microsoft Excel and analysed using STATA statistical analysis software package version 9 (Stata Corp., College Station, TX, USA, 2003). Simple associations were tested using Chi-square test, *t*-test and proportional test where applicable. Regression model was fitted to assess risk of malaria infection considering all possible relevant factors. Effect was considered at 5 % level of significance.

The mRDT sensitivity was estimated as true positives/ (true positives + false negatives), specificity as true negatives/ (true negatives + false positives), positive predictive value as true positives/ (true positives + false positives), negative predictive value as true negatives/ (true negatives + false negatives) and the positive likelihood ratio as sensitivity/ (100 – specificity). Inter-observer variability was assessed using Kappa statistic (with < 0.40 indicating poor to fair agreement, 0.40–0.60 moderate agreement, 0.60–0.80 substantial agreement and > 0.80 almost perfect agreement) while .

The World Health Organization age-adjusted cut-off for haemoglobin (Hb) was used to classify anaemia in children [22]. For children between 5–11 years normal Hb level was defined as Hb ≥11.5 g/dl and mild as 11.0-11.4 g/dl. For children aged 12–15 years, normal Hb level was defined as Hb ≥12 g/dl and mild as 11.0-11.9 g/dl. For both age groups, moderate anaemia was defined as individuals with Hb level of 8–10.9 g/dl while severe anaemia was defined as Hb level of <8 g/dl. At a community level prevalence of anaemia was stated to be severe if over 40 % of the children were anaemic (combining mild, moderate and severe) and moderate if the prevalence was 20–39.9 %. Body Mass Index (BMI) was calculated using the weight and height relationship. BMI was categorized into three groups such as underweight (<18.50 kg/m^2^), normal weight (18.50-24.99 kg/m^2^) and overweight (≥25.00 kg/m^2^). Temperature readings were categorized into fever and non-fever status with a cut-off point of 37.5 °C.

### Ethical considerations

This study received an ethical approval from the Medical Research Coordinating Committee of the National Institute for Medical Research. Before the survey began, the objectives, methods and benefits of the study were explained to the district authority, school teachers and parents in a meeting convened by the village authority. An informed oral consent was sought from parents/guardians of the children involved in the study. An oral assent was asked from each child enrolled in the study. Any child found to be infected with malaria was treated using artemether-lumefantrine tablets according to National Guidelines on Malaria Treatment.

## Results

### Prevalence of malaria

A total of 1,019 school children (mean age = 9.4 ± 2.2 years) were examined for malaria infection. Of the children, females accounted for 54.6 %, while males accounted for 45.4 % (Table 1). *Plasmodium falciparum* was the only (100 %) malaria parasite species observed. The overall prevalence of malaria infection was 10.6 % by mRDT and 4.5 % by microscopy (Table 2). The malaria prevalence in rice irrigation villages was significantly higher (18.2 %) than in pastoral villages (3.0 %) (*p*-value < 0.001). Within the rice irrigation ecosystem, malaria prevalence was higher in Malui (22.5 %) than in Tindiga (13.8 %) (Two-sample test of proportion, *p*-value = 0.005). There were no significant difference in malaria prevalence between villages within the pastoral villages (3.8 % in Mbwade and 2.2 % in Twatwatwa; Two-sample test of proportion, *p*-value = 0.28). Only three (0.23 %) children were found with gametocytes (Tindiga = 1, Malui =1 and Twatwatwa =1). Six (0.6 %) of the children had an enlarged spleen; and all were from rice irrigation villages.

**Table 1:** Demographic characteristics of the children in Kilosa District by village

Variable		Malui	Mbwade	Tindiga	Twatwatwa	Total
Sex	Female	155 (60.1)	125 (52.3)	137 (54)	139 (4.2)	556 (54.6)
	Male	103 (39.9)	114 (47.7)	116 (46)	130 (45.8)	463 (45.4)
Mean age in years (±SD)		9.8 (2.5)	9.9 (2.6)	8.3 (1.8)	9.6 (1.9)	9.4
Age groups (in years)	4-7	48 (18.6)	55 (23.01)	95 (37.5)	38 (14.13)	236 (23.2)
	8-12	164 (63.6)	146 (61.1)	152 (60.1)	216 (80.3)	678 (66.5)
	13-16	46 (17.8)	38 (15.9)	6 (2.4)	15 (5.6)	105 (10.3)
	Total	258	239	253	269	1019

**Table 2:** Prevalence of malaria infection and splenomegaly by livelihoods and village in Kilosa district

Ecosystem	Village	N	mRDT	Microscopy	Splenomegally	Use of net
			N (%)	N (%)	N (%)	(%)
Rice irrigation	Tindiga	253	35 (13.8)	22 (8.7)	4 (1.6)	83.2
	Malui	258	58 (22.5)	20 (7.8)	2 (0.8)	86.2
Dry savannah	Twatwatwa	269	6 (2.2)	2 (0.7)	0 (0.0)	75.6
	Mbwade	239	9 (3.8)	2 (0.8)	0 (0.0)	80.7
	Total	1,019	108 (10.6)	46 (4.5)	6 (0.6)	83.2

Key: N = number of children screened

In all the four villages, higher malaria prevalence was detected by the use of mRDTs. The prevalence of malaria infection by microscopy was 2.4 lower than that found using mRDT. Among the 46 individuals who tested positive by microscopy, 17.4 % were found negative by mRDT. Amongst those tested positive by mRDT only 34 % were found infected with malaria parasites using microscopy. The mRDT specificity and sensitivity were 94.18 % and 82.61 %, respectively. Inter-observer variability between the results of the two tests gives a Kappa value of 0.45 (*p*-value <0.001) indicating moderate agreement.

### Attributes of risk of malaria infection

A multivariate regression model was fitted to assess factors related to the prevalence of malaria. No significance difference in malaria prevalence was observed between sex, age or possession of mosquito net. However a reduction on the odd of acquiring malaria infection was observed for those who were using mosquito nets. Children over 8 years old were observed to be at higher risks of acquiring malaria infection as compared to the younger children. A significant high risk of malaria was observed among children from the rice irrigation villages (Table 3).

**Table 3:** Odd ratio of malaria prevalence in relation to sex, age, livelihood and presence of a health facility

Variable	Odd ratio	[95 % CI]	*P*-value
Sex (Male = 1)	1.35	0.89, 2.05	0.15
Net use (Yes = 1)	0.63	0.37, 1.05	0.07
Age (Reference: 13-16years)			
4-7 years	0.99	0.61, 1.62	0.975
8-12 years	1.47	0.69, 3.13	0.315
Ecosystem (Reference: rice irrigation farming)			
Pastoral community	0.13	0.07, 0.23	<0.001
Presence of health facility (HF available = 1)			
Health facility available	0.55	0.35,0.86	0.01

Presence of a health facility showed an effect on the risk of malaria infection. Those living in villages with health care facilities had low odd of malaria infection by 45 % (OR: 0.55; 95 % CI. 0.35-0.86). Comparing between the two villages in the rice irrigation ecosystem, the prevalence of malaria was 38.5 % higher in Malui (without health facility) than in Tindiga (with health facility).

**Fig. 2:**
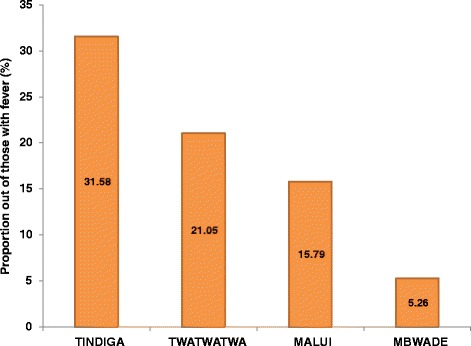
Prevalence of fever (≥37.5 °C) among children in Kilosa District by village

There were 14 children (1.4 %) with fever (temperature >37.5 °C). Ten (71.4 %) of them were from villages with health facilities. Among them, six (42.9 %) were from Tindiga; others were from Twatwatwa (28.6 %), Malui (21.4 %) and Mbwade (7.1 %). A total of 12 children with fever had no malaria infection. The proportion of those with fever but no malaria infection was highest in Tindiga (1.75 %) and lowest in Mbwade (0.42 %). The proportions of children with fever in the other villages were 1.28 % in Malui and 1.58 % in Twatwatwa (Fig. 2).

### Anaemia and nutritional status

**Fig. 3:**
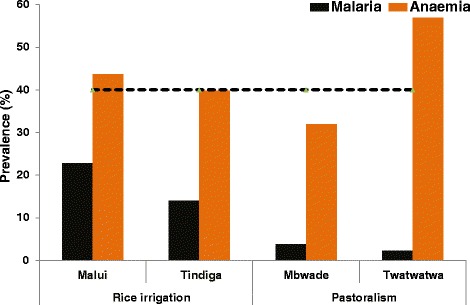
Prevalence of anaemia and malaria by village and livelihoods in Kilosa, Tanzania (The dotted line shows a public health significance cut-off point for prevalence of anaemia)

Overall, the prevalence of anaemia in the district was 43.4 % (n = 775). Schoolchildren from the pastoral community of Twatwatwa village had the highest prevalence of anaemia (Fig. 3). Amongst those with anaemia, 42.3 % (n = 328) had mild anaemia, 55.2 % (n = 428) had moderate and 2.5 % (n = 19) had severe anaemia. Over half (58.3 %) of those with severe anaemia were children among the pastoral communities (33 % Mbwade, 25 % Twatwatwa). No difference in the prevalence of anaemia was observed between female and male children. Anaemia was significantly higher among children not using mosquito nets (45.6 % vs. 39.6 %, *p*-value = 0.049); and among those with malaria infection (57.3 % vs. 39.1 %; *p*-value < 0.001).

The overall mean body mass index (BMI) was 14.9 kg/m^2^ (minimum = 10.6; maximum = 22.7 kg/m^2^) indicating severe thinness. The majority (96.6 %) of the children had BMI less than 18.5 kg/m^2^ which indicate high proportion of underweight. Only 3.4 % had normal weight and most of these (52.9 %) were from Malui village. Tindiga had the highest proportion (98.8 %) of children with underweight. The proportions of underweight in the other villages were 92.9 % in Malui, 96.6 % in Mbwade and 98.1 % in Twatwatwa. There was no significant difference in individuals with normal weight between those from rice and pastoral communities (*p*-value = 0.07).

## Discussion

The findings of this study indicate that children from rice irrigation villages had higher prevalence of malaria than their counterparts in the pastoral villages. Higher malaria prevalence among communities living in rice irrigation ecosystems have been reported elsewhere in Tanzania and in Sub-Saharan Africa [5, 23, 24]. Flooded paddy fields are known to provide ideal breeding sites for *Anopheles gambiae* [23] and hence increase the annual duration of transmission [14]. The variation between pastoral and rice irrigation farming community is likely to have been attributed to the ecological and livelihoods factors. The pastoral community villages are characterised by dry savannah vegetation with minimum potential breeding sites, low malaria mosquito densities transmission and hence low transmission indices [25]. Similar findings have been reported from studies in Mvomero and Moshi districts in Tanzania where the prevalence of malaria in savannah villages was relatively lower than in the rice-irrigation villages [5, 26].

Our results also indicate significant variations in the level of parasitaemia even between villages within the same agro-ecosystem. This variation was attributed to availability of health care facility. The fact that malaria prevalence was higher among children living in villages without health facilities confirms previous findings elsewhere in Tanzania [16, 17]. It is likely that individuals who live closer to the health facilities have better access to health care services than those living far from health facilities. In another study involving the same villages in Kilosa district, the level of knowledge on malaria was significantly associated with education level of the respondents and availability of health care facility in the village [27]. These two factors are therefore likely to contribute to the differences in malaria prevalence observed.

Although there was no significant difference in malaria prevalence between sex, age or possession of mosquito net, the likelihood of acquiring malaria infection was lower among those who were using mosquito nets. This indicates the protective effect of the nets. In this study, it was noted that a high proportion of children with fevers were from villages with health facilities. In addition, the observation that a proportion of children with fever had no malaria infection indicates that non-malaria fevers are prevalent in the area. Fevers of bacteria or viral origin are prevalent in Tanzania and are the most likely causes of febrile illnesses in most cases [28]. In a recent study in Kilosa district, Chikungunya and Dengue fever viruses were diagnosed among patients with febrile illnesses attending health care facilities [29].

In this study the mRDT specificity and sensitivity were high. Other studies in Tanzania have also reported similar higher sensitivity and specificity of histidine-rich protein-2 mRDTs [30, 31]. On the other hand, while one study in north-western Tanzania reported a low sensitivity (29.8 %) but a high specificity (98.8 %) [32], a study in south-eastern Tanzania has reported a high sensitivity (96.1 %) but a slightly low specificity (63.1 %) [33].

Anaemia prevalence in the district was 43.4 %, with the highest prevalence among children in the pastoral villages. Similar high prevalence of anaemia has been observed in the neighbouring district of Mvomero [5]. In this study, anaemia was most prevalent among communities with low malaria prevalence suggesting that the anaemia in the pastoral communities was most likely to be the result of dietary deficiency or parasitic infections other than malaria. Discussion with a number of local leaders among the pastoralists [14] revealed that when grazing reserves are exhausted, they move longer distances in search of forage and water, leaving behind children and old persons with inadequate food reserve. Usually, those left behind experience food insecurity and suffer from malnutrition [34]. Anaemia is a major health problem in Tanzania, especially among children [35]. In a nation-wide survey in 2004/2005, the most common cause of anaemia among children in Tanzania was identified to be nutritional anaemia resulting from inadequate dietary intake of nutrients [36].

The majority of the children in this study were underweight, with most of them from rice growing communities, where malaria prevalence was higher. Some reports already indicate that underweight among children in developing countries is due to lack of food and the presence of diseases, mostly malaria [37]. In a study in western Kenya, a significant association between malaria and nutritional status in children was demonstrated to be associated with higher parasitaemia [38]. Studies in Nigeria and Haiti have shown that low body mass index is associated with a significance increase in childhood malaria [39, 40]. These two studies are in support of the view that malnutrition is associated with increased occurrence of infection and symptomatic malaria [41]. Despite these facts, the relationship between malaria and malnutrition remains unclear and controversial. While malnutrition appears to influence susceptibility to malaria and affects the course of the infection, malnutrition is said to protect against malaria infection [42]. While a study in Colombia reported a log-linear increase in malaria parasite density [43], a recent study in Uganda has shown that severe food insecurity may increase the risk for malaria infection [44].

In this study, strong effect of the ecosystem, livelihoods and health systems on malaria was observed. Rice farming communities were at higher risk of malaria as a result of their routine farm activities that involves irrigation that supports mosquito productivity [4]. In a parallel study in the same villages, it was observed that human biting rate per person per night of malaria mosquitoes was highest in rice irrigation villages than pastoral villages [25]. Farming activities including planting and weeding during the rainy season and once the rice begins to grow, warding off wild birds and animals throughout the ripening stage, tends to expose these communities to mosquito bites and hence high risk of malaria infection. In addition, the agricultural calendar necessitates the adults to move back and forth between the villages and the paddy fields or sometimes even migrate temporarily to the fields, often with families without mosquito prevention tools [14]. A longitudinal study is therefore recommended to establish seasonal patterns of the relationship between malaria prevalence, livelihood, food insecurity, and nutritional status.

## Conclusions

The findings of this study indicate that in Kilosa district, there are variations in terms of risk to malaria, anaemia and nutritional statuses between communities living in different ecosystems and with different livelihoods practices. The variability of the prevalence of malaria parasitaemia by ecosystems and livelihoods illustrates the existing of different micro-ecological zones and the contribution of human activities on transmission factors. It is therefore important, that malaria control measures are designed taking into consideration the specific local conditions and needs. The links between malaria, ecosystem, livelihoods and food insecurity needs to be addressed in tandem using an ecohealth approach.
